# Active Transport to School May Reduce Psychosomatic Symptoms in School-Aged Children: Data from Nine Countries

**DOI:** 10.3390/ijerph17238709

**Published:** 2020-11-24

**Authors:** Dorota Kleszczewska, Joanna Mazur, Jens Bucksch, Anna Dzielska, Catherina Brindley, Agnieszka Michalska

**Affiliations:** 1 Institute of Mother and Child Foundation, 01-211 Warsaw, Poland; 2 Collegium Medicum, University of Zielona Góra, 65-046 Zielona Góra, Poland; joanna.mazur@hbsc.org; 3 Department of Child and Adolescent Health, Institute of Mother and Child, 01-211 Warsaw, Poland; anna.dzielska@imid.med.pl; 4 Department of Natural and Sociological Sciences, Heidelberg University of Education, 69120 Heidelberg, Germany; bucksch@ph-heidelberg.de (J.B.); brindley@ph-heidelberg.de (C.B.); 5 Department of Biomedical Foundations of Development and Sexology, Faculty of Education, University of Warsaw, 01-211 Warsaw, Poland; michalska.agg@gmail.com

**Keywords:** physical activity, wellbeing, active transport, youth, school, mental health, psychosomatic complaints

## Abstract

It is widely proven that being physically active and avoiding sedentary behaviour help to improve adolescents’ well-being and keep them in better health in general. We aimed to investigate the relationship between modes of transport to school and subjective complaints among schoolchildren. Analyses were based on the HBSC (Health Behaviour in School-aged Children) surveys conducted in 2017/18 in nine countries (N = 55,607; mean age 13.43 ± 1.64 yrs.). The main outcome showed that health complaints consisted of somatic and psychological complaints. Transport to school was characterized by mode of getting there (walking, biking, or another passive mode). A total of 46.1% of students walked and 7.3% cycled to school; 46.6% commuted by passive means. Biking to school was more frequent in Denmark (37.9%), Norway (26.5%), and Germany (26.6%). The multivariate generalized linear model adjusted for age, gender, country, and school proximity showed that biking to school is protective against reports of health complaints. The beta parameters were equal to −0.498 (*p* < 0.001) for the general HBSC-SCL index, −0.208 (*p* < 0.001) for the somatic complaint index, and −0.285 (*p* < 0.001) for the psychological complaints index. Young people who actively commute to school are less likely to report health complaints, especially psychological symptoms.

## 1. Introduction

Improving the health of young people is currently one of the priority areas of public health activities. For this reason, it is worth paying attention to non-specific health complaints, which are lately more often communicated by young people [1]. They are described in the literature as “psychosomatic complaints” and include physical symptoms such as backache, headache, and abdominal pain, as well as mental symptoms including fatigue, irritability, or difficulty falling asleep [2]. Non-specific health complaints may also occur due to unhealthy everyday habits, i.e., sleep deficits [3,4], cigarette smoking, or sitting for too long in front of a TV or computer screen [5].

A physically active life and avoiding long episodes of sedentary behaviour help prevent non-specific health complaints and improve well-being among adolescents [6,7,8]. A study involving over 171,000 teenagers from 37 countries revealed that young people who lead physically active, healthy lifestyles experience psychosomatic symptoms less frequently [9]. In particular, the protective functions of physical activity are well established in the context of mental health [10]. Physical activity goes far beyond sports. According to WHO recommendations, moderate to intense physical activity is important, including daily activities that require energy expenditure, such as cleaning, walking upstairs, or active transport to school (ATS) [11]. A key to changing to a more active lifestyle is to implement more physical activity into daily life, including active travel, which does not require financial costs or making huge changes in one’s course of everyday life. [12]. Over the last two decades, studies have shown that ATS plays a significant role in improving adolescent health in many countries [13,14,15]. Existing studies have focused mainly on walking and cycling as two forms of active transport [16]. Cycling to school enables young people to meet the WHO recommendations for daily physical activity to a greater extent [17], reduces the risk of obesity [18], improves overall body fitness, and ensures proper blood circulation [19]. ATS also strengthens positive effects on mental health, helping students perform better in school. This positive effect on mental well-being is significant, as is the joy that physical activity generates and the contact with nature while walking or cycling. It is assumed that ATS can help one build a sense of independence and self-confidence as well as concern for the environment [20].

However, ATS has changed throughout the last decades. Several decades ago, walking to school was the norm in most societies [21], while in more economically developed countries today, children are usually “chauffeured” or driven to school in their parents’ cars [22]. A British study showed a 9% drop in walking to school in 1975–1994 among children aged 5–10 [23]. A similar tendency was recorded in Switzerland, indicated by a 7% decrease in ATS rates among children aged 6–14 between 1994 and 2005 [24]. A 20% decrease in ATS rates was recently recorded in Czechia among adolescents aged 11–15 years [25]. However, a gender difference is well established in active travel, showing that boys are more likely to walk or cycle to school than girls [26]. Moreover, there is a clear link between age and the declining prevalence of ATS [27]. Furthermore, ATS seems to vary by socio-ecological determinants, as patterns of ATS differ between countries according to geographical and cultural backgrounds [26]. Finally, when analysing ATS, the distance to school is one of the strongest predictors for the likelihood of undertaking ATS [28].

When analysing the international literature, we were not aware of any study on the relationship between modes of transport to school and psychosomatic complaints of adolescents in international samples. This contribution refers to data from the international HBSC (Health Behaviour in School-aged Children) study. The HBSC Symptom Check List (HBSC-SCL) has been part of the HBSC questionnaire since the beginning. Gender and age-related demographic determinants and selected psychosocial and behavioural correlates, including those related to physical activity, have been extensively discussed [4,5,7,10]. To our knowledge, no research on the protective effect of active transport to school has been conducted so far based on HBSC data. The authors of this paper assume that this could be a valuable contribution in this matter. We examine the relationship between non-specific somatic complaints among adolescents and ATS for nine European countries of various geographical backgrounds. We hypothesize that ATS is significantly associated with psychosomatic complaints in adolescents and that the protective effect of cycling is greater than that of other forms of ATS, including walking. The aim of the study was to investigate the relationship between modes of transport to school and subjective complaints among schoolchildren. We have set the following research questions:

1. Are there cross-country differences in terms of active transport to and from school?
2. Do young people who use active forms of transport to school report psychosomatic complaints less frequently?
3. Does cycling reduce the incidence of psychosomatic complaints in adolescents more than other forms of active transport to school?
4. Do difficulties in getting to school by active transport moderate the above relationships (duration of travel)?

## 2. Materials and Methods

The HBSC network provides data on health behaviour and outcomes for children and adolescents from 49 countries over a four-year study period. Our data are derived from the last survey cycle of the international HBSC research carried out in the 2017/18 school year. HBSC has developed as a unique mandatory tool for psychosomatic complaints in children and adolescents. For the 2018 study wave, nine member states optionally collected data on ATS. The international sample of the countries covering the topic of ATS and psychosomatic complaints includes 56,834 individuals from nine countries and regions: Azerbaijan (N = 4582), Czechia (N = 11,553), Denmark (N = 2837), Germany (N = 4147), Ireland (N = 3806), Norway (N = 2759), Poland (N = 5191), Scotland (N = 4892), and Wales (N = 15,840). Surveys were conducted in schools according to a standardized procedure described in the international research protocol [29]. The response rate on the student level varied by country, from 53.7% in Germany to 99.3% in Azerbaijan.

The international sample consisted of 49.3% boys and 50.7% girls. The percentage of boys ranged from 47.3% (Germany) to 50.6% (Ireland). Students were categorized into three age groups: 11-year-olds (35.4%), 13-year-olds (34.6%), and 15-year-olds (30.0%). The mean age was 13.43 years (SD = 1.64) and ranged from 13.05 in Norway to 13.59 in Poland.

### 2.1. Variables

#### 2.1.1. Psychosomatic Complaints

Psychosomatic complaints were self-reported. The HBSC Symptom Checklist (HBSC-SCL) includes eight symptoms and is a validated scale that has been used for all HBSC study waves throughout the HBSC process [30]. The adolescents reported how often they had experienced particular complaints in the last 6 months according to a 5-category response scale (from rarely or never to almost daily), coded from 0 to 4. The total (HBSC-SCL) scale range is 0–32 points. We also created two independent indexes: (1) somatic complaints (HBSC-SCL_S) including headache, abdominal pain, back pain, and dizziness, and (2) psychological complaints (HBSC-SCL_P) including depression, nervousness, irritability or bad mood, and difficulty sleeping. These both had index ranges of 0–16 points. Higher scores on individual scales represent greater psychosomatic complaints. In the combined international sample, the HBSC-SCL index has a single-factor structure with reliability of α = −0.804. The HBSC-SCL_S and HBSC-SCL_P indexes are also homogeneous and have a reliability of 0.673 and 0.744, respectively. The percentage of missing data was 3.8% for the entire HBSC-SCL scale and for subscales was 2.8% (HBSC-SCL_S) and 3.4% (HBSC-SCL_P).

#### 2.1.2. Active Transport to School

Students self-reported how they usually get to and from school. There were five mutually exclusive categories of answers: (1) on foot; (2) by bicycle; (3) by bus, train, tram, or metro; (4) by car, motorbike, or scooter; and (5) other means. For this study, three groups of students were identified, creating a single variable based on their answers about getting to school and back home. In the absence of data on a one-way route, students were categorized based on the second type of available information. Individual categories of this variable are:

- walking–if the bicycle was not used and the student usually walked at least one way;
- riding a bicycle–if the bicycle was used at least one way;
- not using active transport–other cases, assumed to be passive ones.

Moreover, the travel to school difficulty index (TSDI), ranging from 1 to 10 points, was defined as an important covariate to control for this effect as the impact of difficulties in active commuting to school which may translate into increased school stress. This index is a measure of the interaction between a typical mode of transport and how long it takes to get to school (estimated only one way). This type of index was assumed to be an approximate measure of the location of the school in relation to home. It illustrates the difficulties with commuting, which may translate into increased school stress. The index ranges from 1 to 10, with “1” indicating a short duration (<5 min) by car and “10” indicating a trip more than 30 min by car. Three experts independently scored the TSDI taking into account the duration of the travel to school and the means of transport. A common version was then discussed and accepted. The TSDI index was created especially for this study. The description of coding rules and definition of the Travel to School Difficulty Index (TSDI) index are given in Table S1 in Supplementary Materials.

### 2.2. Statistical Analysis

To characterize the description of the sample, we used a chi-square test to compare the categories of ATS in groups according to gender, age, and country. The correlation between the symptom indexes and the TSDI variable was examined with the Spearman coefficient.

Kolmogorov–Smirnov test for normality (with Lilliefors correction) indicated that all applied scales were not normally distributed: SCL_T: KS = 0.11, *p* < 0.001; SCL_S: KS = 0.18, *p* < 0.001; SCL_P: KS = 0.13, *p* < 0.001; TSDI: KS = 0.16, *p* < 0.001). So, non-parametric analyses were conducded.

The mean values of the SCL_T, SCP_S, and SCL_P indexes were compared in groups of active and passive commutes to school using a nonparametric Kruskal–Wallis test. Similar calculations for each of the nine countries are provided as supplementals.

We used a generalized linear model (the GENLIN procedure of the SPSS software, IBM SPSS Statistics for Windows, Version 25.0, IBM Corp: Armonk, NY, USA) in the multivariate analysis and included gender, age group, country, or region and the predominant way of reaching school according to the three predefined categories as predictors of successive complaint indexes. The reference categories were as follows: 15-year-olds, girls, students from Azerbaijan, and young people not using active forms of transport from or to school. We also estimated models specific to the nine countries, presenting the values of beta parameters at levels related to active transport (walking, biking).

## 3. Results

### 3.1. Prevalence of Psychosomatic Complaints

The burden of non-specific psychosomatic complaints is presented in Table 1. In the total sample of students from three grades, the average overall symptom index (HBSC-SCL) was 8.28 ± 6.52 (median 7.00) and ranged from 5.64 in Azerbaijan to 9.15 in Poland. Similarly, the two sub-indexes (HBSC-SCL_S and HBSC-SCL_P) had the lowest values in Azerbaijan and the highest in Germany and Poland. Girls reported psychosomatic complaints significantly more often than boys. This applies to the general index and its two domains. In the international sample, the mean HBSC-SCL indexes were 7.12 ± 5.88 for boys and 9.39 ± 6.90 for girls (*p* < 0.001). Significant differences among girls were noted in all countries except Azerbaijan. The lack of gender-related differences in that country mainly concerned the HBSC-SCL_S subscale (*p* = 0.270), because in the case of the HBSC-SCL_P sub-scale, the results were already significant, although at the border of statistical significance (*p* = 0.044).

There was also a clearly increasing burden of psychosomatic symptoms in subsequent age groups. The mean overall HBSC-SCL_T index was as follows: 11-year-olds, 7.00 ± 5.97; 13-year-olds, 8.40 ± 6.54; and 15-year-olds, 9.56 ± 6.80 (*p* < 0.001). Age-related differences were statistically significant in all nine countries. This was confirmed for the overall HBSC-SCL_T index as well as the HBSC-SCL-S and HBSC-SCL-P sub-indexes (data not shown).

### 3.2. Active Transport to School

In total, almost half of the respondents (46.6%) reported using passive forms of commuting to school. The results of the chi-sq test indicate that there is a statistically significant association between countries and active transport to school (chi-sq (1, N = 55,607) = 14,942.61, *p* < 0.001). The proportion ranged from 16.5% in Azerbaijan to 68.4% in Ireland. Almost the same number of students reported walking to school, as the proportion was 46.1%, ranging from 25.4% in Denmark to 81.2% in Azerbaijan; 7.3% of students reported using a bicycle as a means of transport. Students in Denmark (37.9%), Norway (26.5%), and Germany (26.6%) were more likely to cycle to school than young people from other countries (Table 2). The difference between countries is significant at *p* < 0.001 (chi-sq = 14,942.6; df = 16).

The total sample showed an association between the gender and age of respondents and the use of active transport on the way to school. Girls use active forms less often, and boys ride bicycles more frequently (chi-sq (2, N = 55,607) = 325,12, *p* < 0.001). Moreover, 11-year-old students are also more active on their way to school than the two older age groups. The frequency of using a bicycle as a means of transport decreases with age (chi-sq (4, N = 55,256) = 223.90, *p* < 0.001).

On the TSDI, ranging from 1 to 10, the surveyed young people assessed the level of complexity of reaching their schools at 4.41 points on average (SD = 2.00). As presented in Table 2, the TSDI values ranged from 3.78 in Ireland to 4.97 in Germany (F(8,55400) = 139.12; *p* < 0.001)). Mean results were similar for boys and girls (4.42 vs. 4.4; t(55407) = 0.999; *p* = 0.318). However, their values were higher in the two older age groups (13- and 15-year-olds) as compared to the 11-year-olds (13-year-olds: 4.07 ± 1.94, 4.55 ± 2.00, and 4.66 ± 2.02, respectively; F(2,55066) = 456.40; *p* < 0.001).

### 3.3. Psychosomatic Complaints in Relation to Mode of Transport to School

In total, the highest values of the general index HBSC-SCL and two partial indexes were reported by adolescents who did not use any forms of active transport (Table 3, Table S2). The values of all three indexes were lower in the group of people walking to school and were the lowest in the case of cycling. For the general index (SCL_T) and that of psychological complaints (SCL_P), differences were found depending on the modes of AST in eight countries (except Scotland), while for SCL_S, differences were significant in six countries (Azerbaijan, Czechia, Denmark, Ireland, Germany, and Norway).

The level of difficulty in getting to school measured by the TSDI correlated significantly with the indexes of complaints, but the values of respective Spearman’s correlation coefficients were low (for HBSC-SCL, rho = 0.070; for HBSC-SCL_S, rho = 0.066, and for HBSC-SCL_P, rho = 0.061). The only country showing no statistically significant correlation between HBSC-SCL_T and TSDI was Denmark. The highest correlation coefficients of TSDI with HBSC-SCL_T were recorded in Ireland (rho = 0.151), Scotland (rho = 0.107), and Poland (rho = 0.076). Relatively higher values of Spearman’s correlation coefficients were also observed in Ireland in relation to the sub-indexes HBSC-SCL_S (rho = 0.134) and HBSC-SCL_P (rho = 0.137).

Table 4 presents a comparison of groups differing in the mode of AST, taking into account age, gender, country, and the TSDI. The results of the generalized linear model indicate that gender and age are significant predictors of psychosomatic complaints. Using Azerbaijan as the reference category (i.e., the country where young people report subjective complaints the least frequently), HBSC-SCL values increased in the remaining eight countries and were the highest in Poland and Wales. Cycling to school remains a factor in reducing HBSC-SCL values. Walking to school did not significantly affect the variability of HBSC-SCL after adjusting for other factors (*p* = 0.086). An increase in TSDI values by one unit increased the HBSC-SCL value by 0.210.

In analogous generalized linear models estimated for partial indexes (unpublished data), the protective effect of cycling to school was maintained. When the dependent variable was the somatic complaint index, the value of parameter B for the variable relating to cycling was −0.208 (SE = 0.0580) –*p* < 0.001. In the model for mental complaints, the value of parameter B with the variable relating to cycling was −0.285 (SE = 0.0749)–*p* < 0.001. In the first case, walking to school was not found to significantly influence the variability of HBSC-SCL_S (beta = 0.020; *p* = 0.499). In the second case, complaints of a psychological nature (HBSC-SCL_P) increased slightly in the group who reported usually walking (beta = 0.080; *p* = 0.035).

It is also worth mentioning that the protective effect of ATS varies by country. Table 5 shows the beta linear regression coefficients estimated in specific models for nine countries. It includes results adjusted for gender, age, and TSDI modification. The protective effect of cycling to school was significant in four countries (Czechia, Denmark, Germany, and Norway), while the protective effect of walking was only evident in Azerbaijan.

## 4. Discussion

The aim of the study was to examine whether ATS reduces the prevalence of non-specific psychosomatic complaints in adolescents from nine countries or regions of the WHO European region. We analyzed a sample of 56,834 students aged 11–15 surveyed in the HBSC round 2017/18. Adjusted for other factors, cycling showed a protective effect against psychosomatic complaints in Czechia, Denmark, Germany, and Norway, and walking showed a protective association for children and adolescents in Azerbaijan. The analyses of the protective function of active transport highlighted several issues. Above all, it is clear that ATS enhances adolescent health. This supports the main hypothesis of the paper—that ATS is significantly associated with psychosomatic complaints, depending on cross-country differences. Thus, this contribution is part of a wide range of international research on active transport as an important element of describing the current situation regarding the health and activity levels of adolescents [29]. According to our knowledge and the international literature, there have been no studies verifying to what extent active transport is associated with non-specific psychosomatic complaints in adolescents, though many studies have shown a positive association with cardiovascular, weight-, and fitness-related outcomes in adolescents. There are also some studies of adult populations showing protective effects in terms of depression and the relationship between ATS and adolescents’ mental health, defined as well-being [31,32].

ATS, as well as engaging in any physical activity, depends largely on culture, family education, and the promotion of a healthy lifestyle at school and in the community. It is also influenced by the level of socio-economic development of a given country [33]. In countries of higher economic status, such as Germany or Denmark, young people are more likely to use bicycles thanks to better infrastructure. In these countries, young people can use bicycle paths, and schools have designated storage spaces for bicycles [34], enhancing the sense of security. According to our analyses, cycling (not walking to school) has the greatest impact on non-specific somatic complaints in these countries. We might also argue that adolescents in countries with less developed economies walk more often, and therefore, walking to school is not an additional part of overall physical activity and does not show health benefits. A small percentage of teenagers use bicycles, which makes it difficult to estimate health effects of cycling to school among this age group. Walking may be the result of an informed decision or the absence of a public transport network. Azerbaijan turned out to be the only country where walking to school protected against psychosomatic complaints.

In analysing the differences between countries adolescent ATS frequency, it is worth bearing legislation in mind. For example, age limits apply to children going to school or riding bicycles independently. In Poland, a 9-year-old child with parental consent may return home on foot, but by bicycle only if they have a bicycle card, starting at the age of 13. Public transport and school buses provided by municipalities can provide attractive alternatives or leave young people from the countryside with no choice.

An interesting finding of our analyses is the negative relationship between age and the frequency of cycling to school. The decline in this mode of AST in older years may result from the general age-related decline in physical activity, but also from school changes. After moving to a secondary school, students often need to travel to another town or cover a longer distance within a particular city [35]; this finding is in accordance with other studies [18] in terms of the TSDI, which is our measure of interaction between the time needed to get to school and the means of transport. Adding this factor significantly improved the quality of the fit of the models. It was assumed that a difficult, prolonged commute to school intensifies perceived stress, influencing the occurrence of non-specific psychosomatic complaints in adolescents. In many studies based on HBSC data, school stress and general stress are mentioned as the main predictors of SCL values [36]. In the international literature, the distance to school is mentioned mainly as a barrier to physical activity [37,38]. In our models, the SCL burden increases as the correction index increases, therefore showing the opposite association for ATS indicators.

In terms of analysing country differences, it is also worth taking physical conditions into accounts, such as geography, topography, and weather. In some countries, during winter, young people choose only passive modes of commuting [39]. In countries such as Scotland, Wales, and Ireland, cycling does not show a protective association with psychosomatic complaints, even though bicycle use is relatively popular. In Wales, bicycle commuters report more frequent symptoms. Similarly, the association with walking from school in Ireland and Denmark was negative, whereas cycling had a positive effect. One explanation could be the distance to school. However, the shorter the distance to school, the less the commute would affect health.

The mode of active or passive transport to school varies by gender. Girls use active forms of transport less frequently, which may be due in part to their general reluctance to engage in physical activity. In the case of active travel, safety issues also play a role in explaining this difference, since boys are allowed more independent mobility. All the more worrying is the fact that with age, the burden of psychosomatic complaints increases in girls, along with the decrease in physical activity. Potrebny et al. confirmed this in an extensive longitudinal study conducted in 1994–2014 on a sample of teenagers from Norway [40]. They showed that health complaints affect teenage boys to a lesser extent than girls, and that the difference is more pronounced in terms of mental health issues. Teenage girls’ lack of ATS may also be a manifestation of reverse dependency. Adolescent girls are more likely to experience non-specific complaints, which discourage them from engaging in physical activity, including ATS [41]. It can also be assumed that parents more often declare their willingness to drive their daughters to school, being concerned with their safety and bearing in mind their reported worse well-being [42]. Further research should address this gender issue specifically to understand and promote active travel in girls.

One of our most important findings is the greater protective effect of ATS in relation to psychological rather than somatic complaints. This is in line with studies that describe the positive effects of physical activity on the mental health of adolescents [8]. It seems that both cycling and walking to school can build self-esteem based on greater independence. Often, ATS is carried out in a peer group and improves relations with schoolmates, creating opportunities for discussions and joint planning of extracurricular activities [34]. It also should be highlighted that young people who are in better health and in better mood engage in physical activity more often and probably are also more willing to actively commute to school. In these cases, lack of subjective health complaints could be a cause of ATS not an effect [43].

When analysing the issue of ATS, it is important to consider the differences in methodological assumptions of the conducted research. This article uses three simple ATS-related questions available as an optional package in the HBSC protocol. We defined secondary indicators on this basis, affecting the results obtained. The main limitations of the conducted research stemmed from the design of the research tool and not considering other factors. Among the limitations, we highlight the cross-sectional character of the study and the fact that data were self-reported by adolescents. We treated modes of transport as mutually exclusive categories according to the HBSC protocol. In planning the analyses, we adopted a number of simplifications, leaving two forms of active transport: walking and cycling. This approach is used in other studies [44]. The use of other equipment/modes and all mixed forms of movement are summarized in one category. The international literature draws attention to the growing popularity of scooters or skateboards and the emergence of electric devices, which are attractive but result in lower energy expenditure [45]. In some studies, public transport is also treated as a form of physical activity if young people have to cover some part of the way on foot [46]. We added a covariate to our methods, the index of difficulty in getting to school (TSDI). This is our measure of the interaction between the time needed to get to school and the means of transport. It seems that its inclusion is a strength of the paper, counterbalancing the above-mentioned limitations. It should be also mentioned that the response rate in some countries was relatively low, e.g., 53.7% in Germany. This could be considered as a limitation of the present study.

When examining adolescents’ ATS in the context of the reduction of non-specific psychosomatic complaints, one should also bear potential negative effects in mind. Walking or cycling in a city with poor air quality can generate a number of complaints, such as headaches and nausea. Deterioration of well-being can also be aggravated by noise and crowds [47]. Nevertheless, a recent systematic review analysing the health impact of active transportation showed that the positive health effects of active transport outweigh the negative influences of air pollution. In our paper, these negative effects are partially illustrated by the TSDI modification factor.

## 5. Conclusions

Considering the significant burden for students with various somatic or psychological symptoms, demonstrating the beneficial effects of ATS may help to set new directions for carrying out interventions. It would be valuable to take cross-country determinants of active travel to school into consideration while planning such interventions. Young people who cycle to school are less likely to report health complaints, especially psychological symptoms. Research on this subject should take into account the cross-country differences. Promoting cycling seems to be particularly beneficial to psychological health but is not common among the studied countries.

## Figures and Tables

**Table 1:** Mean indices of psychosomatic complaints reported by students from nine countries.

Country /WHO Region	HBSC-SCL ^1^ (n-53491)		HBSC-SCL_S ^2^ (n-54052)		HBSC-SCL_P ^3^ (n-53741)	
	Mean	SD	Mean	SD	Mean	SD
Azerbaijan	5.64	6.87	2.40	3.44	3.22	4.06
Czechia	8.33	5.82	2.66	2.73	5.63	3.89
Denmark	7.80	5.96	2.91	3.05	4.86	3.71
Germany	8.16	5.89	3.58	3.18	4.56	3.52
Ireland	8.32	6.57	3.07	3.22	5.18	4.05
Norway	7.48	5.98	2.72	2.96	4.72	3.69
Poland	9.15	6.48	3.08	3.09	6.02	4.29
Scotland	8.50	6.87	3.15	3.37	5.31	4.26
Wales	8.89	6.91	3.36	3.37	5.49	4.34
Total	8.28	6.52	3.04	3.19	5.20	4.14

^1^ HBSC-SCL–total index of psychosomatic symptoms; ^2^ HBSC-SCL_S–index of somatic symptoms; ^3^ HBSC-SCL_P–index of psychological symptoms.

**Table 2:** Characteristics of commuting to school in nine countries.

Country /WHO Region	Means of Transport (%) (N-55607)			Level of TSDI ^1^ (N-55409)	
	Passive	Walking	Biking	Mean	SD
Azerbaijan	16.5	81.2	2.3	4.37	1.94
Czechia	35.0	62.1	2.9	4.29	1.88
Denmark	36.7	25.4	37.9	4.34	1.81
Germany	54.6	18.9	26.6	4.97	1.94
Ireland	68.4	27.9	3.7	3.78	2.05
Norway	33.8	39.8	26.5	4.52	1.82
Poland	41.1	52.8	6.1	4.32	2.00
Scotland	46.8	51.8	1.4	4.10	1.98
Wales	62.3	36.5	1.2	4.64	2.11
Total	46.7	46.1	7.3	4.41	2.00

^1^ TSDI–transport to school difficulty index.

**Table 3:** Psychosomatic complaints according to mode of transport to school in the combined sample from nine countries.

Transport to School	SCL_T ^1^		SCL_S ^2^		SCL_P ^3^	
	Mean	SD	Mean	SD	Mean	SD
Passive mode	8.57	6.57	3.18	3.24	5.34	4.15
Walking	8.15	6.57	2.94	3.16	5.17	4.20
Biking	7.27	5.72	2.76	2.97	4.48	3.58
Kruskal–Wallis test	-		-		-	
Chi-sq	141.22		133.28		125.52	
df	2		2		2	
p	<0.001		<0.001		<0.001	

^1^ SCL_T–total index of psychosomatic symptoms; ^2^ SCL_S–index of somatic symptoms; ^3^ SCL_P–index of psychological symptoms.

**Table 4:** Generalized linear regression model for total index of psychosomatic symptoms (HBSC-SCL) estimated on the combined sample from nine countries (N-53016).

Parameter	B	SE(B)	Wald Statistics	df	p
(Constant)	6.940	0.1342	2675.525	1	0.000
Age category					
11 yrs	−2.402	0.0682	1238.425	1	0.000
13 yrs	−1.165	0.0674	298.659	1	0.000
15 yrs (ref.)					
Gender					
Boys	−2.252	0.0546	1704.425	1	0.000
Girls (ref.)					
Mode of transport to school					
Walking	0.102	0.0596	2.941	1	0.086
Biking	−0.498	0.1181	17.790	1	0.000
Passive mode (ref.)					
TSDI ^1^	0.210	0.0139	228.058	1	0.000
Country/WHO region					
Czechia	2.702	0.1130	571.464	1	0.000
Denmark	2.542	0.1595	253.785	1	0.000
Germany	2.486	0.1429	302.617	1	0.000
Ireland	2.925	0.1445	409.642	1	0.000
Norway	2.270	0.1579	206.752	1	0.000
Poland	3.534	0.1307	731.153	1	0.000
Scotland	3.027	0.1333	516.126	1	0.000
Wales	3.304	0.1113	882.060	1	0.000
Azerbaijan (ref.)					
(Scale)	39.075	0.2400			

^1^ TSDI—transport to school difficulty index.

**Table 5:** Beta parameters related to the effect of active transport to school estimated by country-specific generalized linear regression models for total index of psychosomatic symptoms (HBSC-SCL).

Country /WHO Region	Effect of Biking			Effect of Walking		
	Beta	SE	p	Beta	SE	p
Azerbaijan	0.190	0.7377	0797	−1.426	0.2783	<0.001
Czechia	−0.669	0.3368	0.047	−0.112	0.1164	0.337
Denmark	−0.593	0.2589	0.022	0.619	0.2864	0.031
Germany	−0.707	0.2109	0.001	0.309	0.2416	0.202
Ireland	−0.142	0.5667	0.802	0.934	0.2346	<0.001
Norway	−0.867	0.3005	0.004	−0.207	0.2664	0.436
Poland	−0.323	0.3839	0.400	0.169	0.1826	0.357
Scotland	0.085	0.8315	0.919	0.234	0.1945	0.230
Wales	1.303	0.5152	0.011	0.327	0.1132	0.004

## Supplementary Materials

The following are available online at https://www.mdpi.com/1660-4601/17/23/8709/s1, Figure S1: Mode of transport to school by age and gender., Table S1. Level of subjective complaints by mode of transport to school.
