# Supplementary material for: Active Transport to School May Reduce Psychosomatic Symptoms in School-Aged Children: Data from Nine Countries
Source: Int J Environ Res Public Health. 2020 Nov 24;17(23):8709. doi: 10.3390/ijerph17238709 (PMC7727647; doi:10.3390/ijerph17238709)
Supplement: Supplementary file 1 [file ijerph-17-08709-s001.pdf]

International Journal of
Environmental Research and Public Health

MDPI

Supplementary Material

Table S1. Level of subjective complaints by mode of transport to school.

Country /WHO region	Passive mode		Walking		Biking		P*
	Mean	SD	Mean	SD	Mean	SD	
			Total index of psychosomatic symptoms (SCL_T)				
Azerbaijan	6.88	7.32	5.36	6.68	6.89	8.81	<0.001
Czechia	8.55	5.88	8.24	5.80	7.40	5.26	0.001
Denmark	8.00	6.06	8.49	6.38	7.14	5.49	<0.001
Germany	8.41	6.02	8.51	6.07	7.41	5.40	<0.001
Ireland	8.13	6.49	8.96	6.84	7.11	5.58	0.001
Norway	8.21	6.31	7.50	5.99	6.52	5.38	<0.001
Poland	9.25	6.51	9.19	6.49	8.17	6.15	0,017
Scotland	8.66	6.90	8.39	6.88	7.01	5.63	0.101
Wales	8.77	6.88	9.10	6.94	8.88	7.60	0.011
			Index of somatic symptoms (SCL_S)				
Azerbaijan	2.91	3.70	2.27	3.33	3.41	4.70	<0.001
Czechia	2.72	2.75	2.64	2.73	2.43	2.51	0,045
Denmark	2.92	3.03	3.23	3.23	2.68	2.91	0.001
Germany	3.76	3.28	3.73	3.18	3.12	2.94	<0.001
Ireland	3.03	3.17	3.29	3.40	2.15	2.49	0.001
Norway	3.07	3.21	2.72	2.91	2.27	2.64	<0.001
Poland	3.12	3.13	3.09	3.07	2.83	3.02	0.241
Scotland	3.22	3.41	3.11	3.33	2.45	3.03	0,091
Wales	3.33	3.36	3.40	3.39	3.66	3.80	0.316
			Index of psychological symptoms (SCL_P)				
Azerbaijan	3.96	4.31	3.06	3.97	3.44	4.75	<0.001
Czechia	5.79	3.92	5.56	3.88	4.94	3.70	<0.001
Denmark	5.04	3.77	5.22	3.96	4.45	3.43	<0.001
Germany	4.64	3.58	4.76	3.69	4.26	3.23	0.004
Ireland	5.03	4.00	5.59	4.18	4.85	3.83	<0.001
Norway	5.09	3.80	4.75	3.74	4.20	3.40	<0.001
Poland	6.08	4.29	6.05	4.30	5.31	4.07	0.009
Scotland	5.40	4.28	5.24	4.25	4.57	3.61	0.239
Wales	5.40	4.32	5.65	4.37	5.19	4.58	0.001

* Kruskal-Wallis test

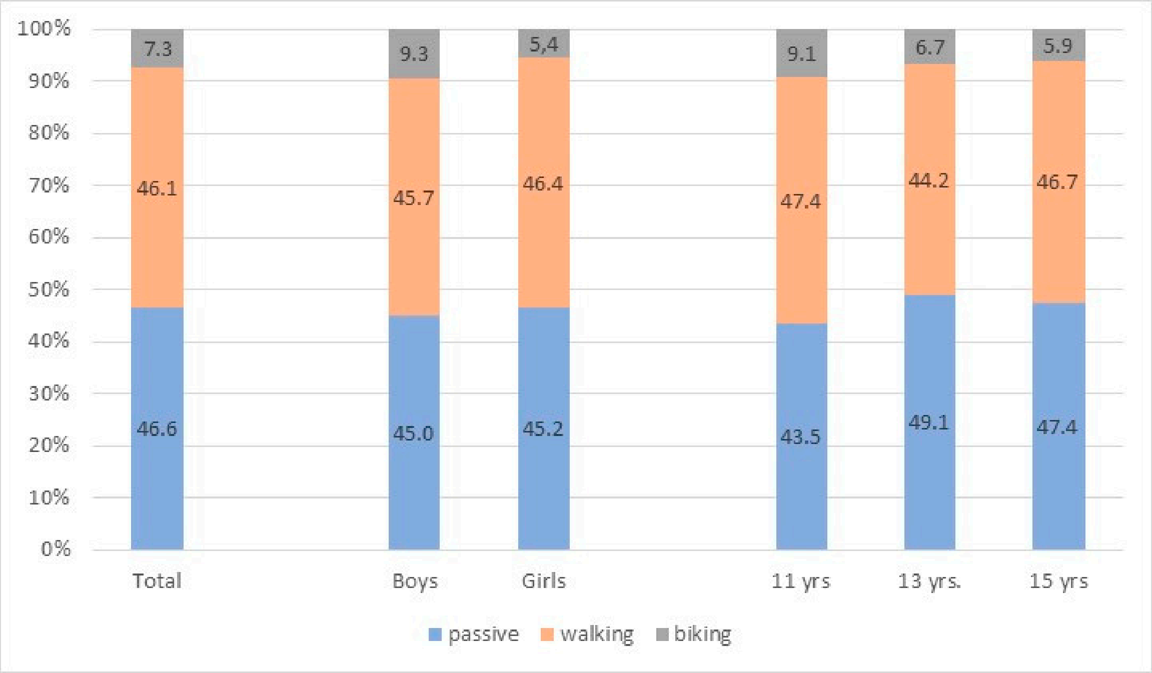

Figure S1. Mode of transport to school by age and gender.
